# Selection Indices and Multivariate Analysis Show Similar Results in the Evaluation of Growth and Carcass Traits in Beef Cattle

**DOI:** 10.1371/journal.pone.0147180

**Published:** 2016-01-20

**Authors:** Fernando Brito Lopes, Marcelo Corrêa da Silva, Cláudio Ulhôa Magnabosco, Marcelo Goncalves Narciso, Roberto Daniel Sainz

**Affiliations:** 1 CNPq Post-doctoral fellow, College of Agricultural & Life Sciences, University of Wisconsin–Madison, Animal Science Building, 1675 Observatory Dr, Madison, 53706, WI, United States of America; 2 CNPq PhD student, Programa de Pós Graduação em Ciência Animal, Universidade Federal de Goiás, Escola de Veterinária e Zootecnia, Campus II, Samambaia, 74001–970, Goiânia, GO, Brazil; 3 Embrapa Cerrados / CNPq, BR 020 Km 18, 73310–970, PO Box: 08223, Planaltina, DF, Brazil; 4 Embrapa Rice and Beans, Rodovia GO-462, km 12, 75375–000—Zona Rural, Santo Antônio de Goiás, GO, Brazil; 5 Department of Animal Science, University of California Davis, Davis, 95616, CA, United States of America; Institute of Farm Animal Genetics, GERMANY

## Abstract

This research evaluated a multivariate approach as an alternative tool for the purpose of selection regarding expected progeny differences (EPDs). Data were fitted using a multi-trait model and consisted of growth traits (birth weight and weights at 120, 210, 365 and 450 days of age) and carcass traits (longissimus muscle area (LMA), back-fat thickness (BF), and rump fat thickness (RF)), registered over 21 years in extensive breeding systems of Polled Nellore cattle in Brazil. Multivariate analyses were performed using standardized (zero mean and unit variance) EPDs. The k mean method revealed that the best fit of data occurred using three clusters (k = 3) (P < 0.001). Estimates of genetic correlation among growth and carcass traits and the estimates of heritability were moderate to high, suggesting that a correlated response approach is suitable for practical decision making. Estimates of correlation between selection indices and the multivariate index (LD1) were moderate to high, ranging from 0.48 to 0.97. This reveals that both types of indices give similar results and that the multivariate approach is reliable for the purpose of selection. The alternative tool seems very handy when economic weights are not available or in cases where more rapid identification of the best animals is desired. Interestingly, multivariate analysis allowed forecasting information based on the relationships among breeding values (EPDs). Also, it enabled fine discrimination, rapid data summarization after genetic evaluation, and permitted accounting for maternal ability and the genetic direct potential of the animals. In addition, we recommend the use of longissimus muscle area and subcutaneous fat thickness as selection criteria, to allow estimation of breeding values before the first mating season in order to accelerate the response to individual selection.

## Introduction

Growth traits are generally useful selection criteria for beef cattle. Weight records at different ages can be easily collected on-farm and tend to present strong correlation estimates [1–3] and moderate to high heritability estimates [4–7]. Weaning weight is usually considered as a correlated trait in the genetic evaluation of livestock and is often used to support culling and selection decisions [8]. Other traits such as fat thickness and muscle area are also fairly easy to measure using real-time ultrasound [9,10], and may furnish accurate data for the estimation of breeding values, as reported for young bulls [10,11].

Expected progeny differences (EPDs) are important tools for animal breeding and selection. However, each EPD reflects an animal’s genetic merit for only a single trait at a time. By contrast, animal breeders dealing with complex production systems must focus on many traits simultaneously. Historically, animal selection has been based on EPDs combined with a portfolio of diversifying approaches (see [12] for tandem selection and selection based on independent culling levels; [13] for index selection; and [14] for an aggregate value index). All methods share the same goal in terms of selecting animals with superior genetic merit for traits of economic importance. Some methods may present advantages which depend on the availability and complexity of data. The selection index approach is time-consuming and is reliant on detailed farm-specific data in order to obtain the economic values of phenotypic traits. These restrictions likely account for the limited application of selection indices, despite proposal of the theory over seven decades ago.

Multivariate analyses are frequently used as an alternative approach for data summarization. In cattle breeding this technique has been applied to efficiently rank and group bulls according to similarity [15]. The k-means method has demonstrated fine clustering of bulls which were submitted to a progeny test [16]. Principal components (PC) analyses are multivariate techniques used mainly to reduce the dimensionality of data and to explore the relationship between traits in a dataset. In animal breeding, PC analysis techniques have been used to study the relationships among the estimated breeding values of various traits [17,18]. Another important multivariate approach also used in quantitative genetic analysis is discriminant analyses [19,20], which also can be used to classify animals based on their breeding values. However, we are unaware of any report attempting to use a multivariate approach for the purpose of genetic selection in cattle. Thus, revisiting the multivariate playground in order to identify and select similar/superior animals using EPDs may prove fruitful. In particular, this approach seems promising for the purpose of accounting for the relationships among breeding values that are related to several traits at the same time. Specifically, multivariate techniques might be suitable for the evaluation of growth and carcass traits, providing an alternative approach in the portfolio of selection tools [18].

This study was carried out using data from Brazil, where the use of EPDs has played a crucial role in increasing livestock productivity. The present status of cattle breeding and selection in this country matches the methodological summary given above. There have been several efforts to estimate covariance and genetic parameters and to predict breeding values in Nellore cattle [3,7,21,22] with no reports on any multivariate approach to selection. Over the past 30 years, Brazilian cattle breeders have widely adopted the use of animal breeding values rather than phenotypes in the selection process.

Currently, Brazil has the world’s second largest commercial beef cattle herd, thus occupying a prominent position in the global beef market. In this country, beef cattle production is expected to increase 5.1% compared to 2014 [23] and Zebu (*Bos indicus*) cattle correspond to more than 80% of the beef-producing herds. The significant variability reported in the evaluation of growth and production traits in Nellore cattle (*Bos indicus*) reared in Brazil seems to mirror the great variability of climate and general management applied in the breeding systems. This is a clear indication that generalization of economic values between herds is unlikely to hold. Conversely, the estimation of economic values for each and every breeding system would be a difficult and time-consuming task. Therefore, an alternative to the economic selection index is needed.

In this study, we propose and evaluate a multivariate approach, restricted to genetic effects, as a strategic practical shortcut for the identification of animals with superior genetic merit.

## Material and Methods

The data set was provided by the National Association of Breeders and Researchers (ANCP) in Brazil and consisted of weights and carcass traits measured between the years of 1995 and 2014. Evaluation of phenotypes took place in a tropical humid region (254 m altitude) in herds located in the county of Pontes e Lacerda, Mato Grosso state, Brazil. Mean annual precipitation is of 1,500 mm with defined wet and dry seasons. Records consisted of birth weight (BW), weights at standard ages of 120 (W120), 210 (W210), 365 (W365) and 450 (W450) days, longissimus muscle area (LMA), back-fat thickness (BF) and rump fat thickness (RF). The following formula generated the weight at standard ages of each animal: *AdjW* = [(*WA* − *WB*)/(*AA* − *AB*)]×(*SA* − *AB*) + *WA*, where *AdjW* is the weight adjusted at standard ages of 120, 210, 365 and 450 days; *WA* and *WB* are the weights before and after standard ages (*SA*), respectively; *AA* and *AB* are ages before and after the standard ages (*SA*), respectively. Adjusted weights were used because all breeding programs in Brazil work with specific selection criteria, mainly weights at standard ages. Records of LMA and BF were obtained from cross-sectional images of the Longissimus muscle, between the 12th and 13th ribs. Back-fat thickness was estimated at the 3/4 position from the chine bone end of the Longissimus muscle using the cross-sectional LMA image. RF was measured over the intersection between the gluteus medium and biceps femoris muscles located between the hooks and pin bones. All ultrasound image collections and analyses were carried out by Ultrasound Guidelines Council-certified technicians, hardware and software.

Exploratory analysis was carried out using the *Statistical Analysis Systems* [24] to check the consistency of the data and to evaluate the significance of environmental sources of variation that can affect the traits, such as current herd, year of birth, season of birth (classified into four groups: Jan-Mar, Apr-Jun, Jul-Sep, Oct-Dec), and sex and cow age at calving, as a covariate. Thus, the contemporary groups (CG) were defined as the groups of animals of the same sex, and born in the same herd, year, and season and reared under the same conditions. Linear and quadratic regressions of the weights by age of dam at calving were performed to determine if this covariate should be included in the genetic analyses. Hence, due to significance (P<0.01) of the dam age at calving, it was used to fit the following traits: birth weight, weight at 120, 210, 365 and 450 days of age. Edits included removal of weights and carcass traits outside of 3 standard-deviations from their respective GC means, discarding contemporary groups of fewer than 9 observations, and discarding contemporary groups represented by a single sire. Summary information of the edited data set is in Table 1. The relationship matrix included 9 generations of pedigree information and contained a total of 28,828 animals.

**Table 1:** Description of the final data set of carcass traits as measured by ultrasound of the live animal, and growth traits in Polled Nelore cattle.

	Trait^a^							
	BW	W120	W210	W365	W450	LMA	BF	RF
**Data structure**								
*No*. *of contemporary groups*	109	113	110	106	103	46	46	46
*No*. *of animals with records*	40119	39883	36228	27626	26153	11676	11665	11621
*No*. *of sires*	555	596	585	580	555	437	435	436
*No*. *of dams*	14189	14190	13489	11350	10694	6316	6314	6300
**Summary statistics**								
*Mean*	32.48	119.24	173.23	221.00	253.74	46.79	1.98	2.43
*Standard Deviation*	3.12	17.12	25.76	32.36	36.30	8.07	0.70	0.82
*Coefficient of Variation (%)*	9.62	14.36	14.87	14.64	14.31	17.24	35.26	33.84

^a^ BW, birth weight (kg); W120, weight (kg) at 120 days of age; W210, weight (kg) at 210 days of age; W365, weight (kg) at 365 days of age; W450, weight (kg) at 450 days of age; LMA: longissimus muscle area (cm^2^); BF: back-fat thickness (mm), obtained between the 12th and 13th ribs; and RF: rump fat thickness (mm)

Genetic analyses were carried out by fitting a model that included the following effects: age of dam as covariate, sex of the animal coded as male or female, season of birth coded into four levels and the effect of the management group on the farm. The GLM (General Linear Model) procedure was used to define the fixed effects included in the contemporary groups. The fixed effects that significantly influenced the growth traits were included in the subsequent analyses. Genetic analysis was done by fitting multi-trait animal models. The mixed linear model for these traits was:

$$y=X\beta +Z_{1a}+Z_{2m}+Z_{3mpe}+e$$

where ***β*** represents the fixed effects (contemporary group as a cross-classified effect) and cow age at calving (as linear and quadratic effects) as covariates), associated with the observation (records), vector ***y*** is the known matrix ***X*** and ***a***, ***m*** and ***mpe*** are the random effects vectors (direct, maternal and maternal permanent environmental effects, respectively) associated with records in ***y*** by the incidence matrix ***Z***_***1***_**, *Z***_***2*,**_ and ***Z***_***3***_, respectively; and ***e*** is the residual (co)variance. For this model, it was assumed that:

$$\mathrm{var}=\left[\begin{array}{c} a\\ m\\ mpe\\ e \end{array}\right]=\left[\begin{array}{cccc} G_{a}⊗A & & & \\ G_{am}⊗A & G_{m}⊗A & & \\ 0 & 0 & E_{mpe}⊗I_{c} & \\ 0 & 0 & 0 & R⊗I_{n} \end{array}\right]$$

Uniform and Gaussian priors were assumed for fixed and random effects, respectively:

$$\begin{array}{l} \beta \propto cons\tan t\;e\\ a|G_{a}∼MVN\left[0,\left(G_{a}⊗A\right)\right]\\ m|G_{m}∼MVN\left[0,\left(G_{m}⊗A\right)\right]\\ mpe|E_{mpe}∼MVN\left[0,\left(E_{mpe}⊗I_{c}\right)\right]\\ e|R∼MVN\left[0,\left(R⊗I_{n}\right)\right] \end{array}$$

where ***G***_***a***_ and ***G***_***m***_ are (co)variance matrices of additive direct genetic effects and additive maternal additive genetic effects, respectively; ***G***_***am***_ is a matrix of additive genetic (co)variances between direct and maternal effects; ***E***_***mpe***_ is a (co)variance matrix of maternal permanent environmental effects; ***R*** is a (co)variance matrix of residual effects; ***A*** is the additive relationship matrix among all animals in the pedigree file; and ***I***_***c***_ and ***I***_***n***_ are identity matrices, whose orders were the numbers of dams and animals, respectively. The inverse *Wishart* distribution was used to derive priors for variance components:

$$\begin{array}{l} G_{a}|S_{a},v_{a}∼IW\left(S_{a}v_{a},v_{a}\right)\\ G_{m}|S_{m},v_{m}∼IW\left(S_{m}v_{m},v_{m}\right)\\ E_{mpe}|S_{mpe},v_{mpe}∼IW\left(S_{mpe}v_{mpe},v_{mpe}\right)\\ R|S_{r},v_{r}∼IW\left(S_{r}v_{r},v_{r}\right) \end{array}$$

where ***S***_***a***_ and ***v***_***a***_, ***S***_***m***_ and ***v***_***m***_, ***S***_***mpe***_ and ***v***_***mpe***_, and ***S***_***r***_ and and ***v***_***r***_ are *a priori* values and degrees of freedom for direct genetic, maternal genetic, maternal permanent environmental and residual covariance, respectively. In matrix notation, the variance structures were:

$$\mathrm{var}=\left[\begin{array}{c} a_{1}\\ a_{2}\\ a_{3}\\ a_{4}\\ a_{5}\\ a_{6}\\ a_{7}\\ a_{8}\\ m_{1}\\ m_{2}\\ m_{3} \end{array}\right]=\left[\begin{array}{cccccccccccc} \sigma_{a_{1}}^{2} & & & & & & & & & & & \\ \sigma_{a_{2}a_{1}} & \sigma_{a_{2}}^{2} & & & & & & & & & & \\ \sigma_{a_{3}a_{1}} & \sigma_{a_{3}a_{2}} & \sigma_{a_{3}}^{2} & & & & & & & & & \\ \sigma_{a_{4}a_{1}} & \sigma_{a_{4}a_{2}} & \sigma_{a_{4}a_{3}} & \sigma_{a_{4}}^{2} & & & & & & & & \\ \sigma_{a_{5}a_{1}} & \sigma_{a_{5}a_{2}} & \sigma_{a_{5}a_{3}} & \sigma_{a_{5}a_{4}} & \sigma_{a_{5}}^{2} & & & & & & & \\ \sigma_{a_{6}a_{1}} & \sigma_{a_{6}a_{2}} & \sigma_{a_{6}a_{3}} & \sigma_{a_{6}a_{4}} & \sigma_{a_{6}a_{5}} & \sigma_{a_{6}}^{2} & & & & & & \\ \sigma_{a_{7}a_{1}} & \sigma_{a_{7}a_{2}} & \sigma_{a_{7}a_{3}} & \sigma_{a_{7}a_{4}} & \sigma_{a_{7}a_{5}} & \sigma_{a_{7}a_{6}} & \sigma_{a_{7}}^{2} & & & & & \\ \sigma_{a_{81}a_{1}} & \sigma_{a_{8}a_{2}} & \sigma_{a_{8}a_{3}} & \sigma_{a_{8}a_{4}} & \sigma_{a_{8}a_{5}} & \sigma_{a_{8}a_{6}} & \sigma_{a_{8}a_{7}} & \sigma_{a_{8}}^{2} & & & & \\ \sigma_{m_{1}a_{1}} & \sigma_{m_{1}a_{2}} & \sigma_{m_{1}a_{3}} & \sigma_{m_{1}a_{4}} & \sigma_{m_{1}a_{5}} & \sigma_{m_{1}a_{6}} & \sigma_{m_{1}a_{7}} & \sigma_{m_{1}a_{8}} & \sigma_{m_{1}}^{2} & & & \\ \sigma_{m_{2}a_{1}} & \sigma_{m_{2}a_{2}} & \sigma_{m_{2}a_{3}} & \sigma_{m_{2}a_{4}} & \sigma_{m_{2}a_{5}} & \sigma_{m_{2}a_{6}} & \sigma_{m_{2}a_{7}} & \sigma_{m_{2}a_{8}} & \sigma_{m_{2}m_{1}} & \sigma_{m_{2}}^{2} & & \\ \sigma_{m_{3}a_{1}} & \sigma_{m_{3}a_{2}} & \sigma_{m_{3}a_{3}} & \sigma_{m_{3}a_{4}} & \sigma_{m_{3}a_{5}} & \sigma_{m_{3}a_{6}} & \sigma_{m_{3}a_{7}} & \sigma_{m_{3}a_{8}} & \sigma_{m_{3}m_{1}} & \sigma_{m_{3}m_{2}} & \sigma_{m_{3}}^{2} & \end{array}\right]⊗A$$

for the direct (u) and maternal (m) effects;

$$\mathrm{var}=\left[\begin{array}{c} mpe_{1}\\ mpe_{2}\\ mpe_{3} \end{array}\right]=\left[\begin{array}{ccc} \sigma_{mpe_{1}}^{2} & & \\ \sigma_{mpe_{2}mpe_{1}} & \sigma_{mpe_{2}}^{2} & \\ \sigma_{mpe_{3}mpe_{1}} & \sigma_{mpe_{3}mpe_{2}} & \sigma_{mpe_{3}}^{2} \end{array}\right]⊗I_{c},$$

for maternal permanent environmental effects (*mpe*); and

$$\mathrm{var}=\left[\begin{array}{c} e_{1}\\ e_{2}\\ e_{3}\\ e_{4}\\ e_{5}\\ e_{6}\\ e_{7}\\ e_{8} \end{array}\right]=\left[\begin{array}{cccccccc} \sigma_{e_{1}}^{2} & & & & & & & \\ \sigma_{e_{2}e_{1}} & \sigma_{e_{2}}^{2} & & & & & & \\ \sigma_{e_{3}e_{1}} & \sigma_{e_{3}e_{2}} & \sigma_{e_{3}}^{2} & & & & & \\ \sigma_{e_{4}e_{1}} & \sigma_{e_{4}e_{2}} & \sigma_{e_{4}e_{3}} & \sigma_{e_{4}}^{2} & & & & \\ \sigma_{e_{5}e_{1}} & \sigma_{e_{5}e_{2}} & \sigma_{e_{5}e_{3}} & \sigma_{e_{5}e_{4}} & \sigma_{e_{5}}^{2} & & & \\ \sigma_{e_{6}e_{1}} & \sigma_{e_{6}e_{2}} & \sigma_{e_{6}e_{3}} & \sigma_{e_{6}e_{4}} & \sigma_{e_{6}e_{5}} & \sigma_{e_{6}}^{2} & & \\ \sigma_{e_{7}e_{1}} & \sigma_{e_{7}e_{2}} & \sigma_{e_{7}e_{3}} & \sigma_{e_{7}e_{4}} & \sigma_{e_{7}e_{5}} & \sigma_{e_{7}e_{6}} & \sigma_{e_{7}}^{2} & \\ \sigma_{e_{8}e_{1}} & \sigma_{e_{8}e_{2}} & \sigma_{e_{8}e_{3}} & \sigma_{e_{8}e_{4}} & \sigma_{e_{8}e_{5}} & \sigma_{e_{8}e_{6}} & \sigma_{e_{8}e_{7}} & \sigma_{e_{8}}^{2} \end{array}\right]⊗I_{n},$$

residual effects.

(Co)variance components were estimated by a Bayesian implementation via Gibbs sampling using flat improper priors for nuisance parameters and flat priors for (co)variance components. The marginal posterior distribution for each parameter was obtained by integration of multivariate density functions, considering one long chain with 1,500,000 iterations. The initial discard was 500,000 and the thinning interval of the chain was 100. Convergence was checked by visual inspection of the sample trace plots. Computations of direct genetic ($\sigma_{a}^{2}$), maternal genetic ($\sigma_{m}^{2}$), covariance between the direct and maternal genetic (*σ*_*am*_), maternal permanent environmental ($\sigma_{mpe}^{2}$) and residual variances ($\sigma_{e}^{2}$) were carried out using the program GIBBS2F90 [25]. The following (co)variance components and parameters were calculated as in [26]: phenotypic variance for weights ($\sigma_{p}^{2}=\sigma_{a}^{2}+\sigma_{m}^{2}+\sigma_{am}^{}+\sigma_{pe}^{2}+\sigma_{e}^{2}$), carcass traits ($\sigma_{p}^{2}=\sigma_{a}^{2}+\sigma_{e}^{2}$), direct heritability ($h_{a}^{2}=\sigma_{a}^{2}/\sigma_{p}^{2}$), maternal heritability ($h_{m}^{2}=\sigma_{m}^{2}/\sigma_{p}^{2}$), genetic correlation between the direct and maternal effect (*r*_*am*_) and maternal permanent environmental variance as a proportion of the phenotypic variance

$$(c^{2}=\sigma_{pe}^{2}/\sigma_{p}^{2}).$$

The expected progeny differences were rescaled to have zero (0) mean and unit (1) variance, and cluster analyses were performed using the k-mean method, which is effective to detect initial clusters with a standard iterative algorithm for minimizing the sum of squared distances from cluster means. A set of points named cluster seeds were selected as a first guess for cluster means. Each animal was assigned to the nearest seed to form temporary clusters. The seeds were then replaced by the means of temporary clusters and the process was repeated until no further changes occurred in the clusters. Therefore, the animals were encoded according to their respective group and k was chosen according to the pseudo F statistic [27], as well as sum of squares within groups. The *dunn*.*test* package [28] was used in order to compute Dunn's test [29] among multiple pairwise comparisons after a Kruskal-Wallis test among k groups. False Discovery Rate (FDR) was controlled using the Benjamini-Yekutieli adjustment [30].

Principal component and discriminant analyses of EPDs were performed in order to use both the PC and the coefficients of linear discriminants (CLD) as a selection index. The proportion of trace was considered in order to determine which CLD should be used, which indicates for how much (as a proportion) of the dataset of standardized EPDs each of the equations can successfully account. The results obtained using the CLD and PC indices were compared to results using a selection index, which was estimated according to [13]. In matrix notation this selection index would be *I* = *b'X*, where *X* is an *n x 1* vector of selection criteria and *b* is an *n x 1* vector of weighting factors to be computed. Thus, the selection index weights were calculated as *b* = *P*^−1^*G*_12_*v*, where *G*_*11*_ is an *n x n* genetic (co)variance matrix of the *n* selection criteria, *P* is an *n x m* phenotypic (co)variance matrix of the selection criteria, and *v* is a vector of economic values. As it is just a simulated index, it was considered that all economic weights (***v***) had the same value (***v*** = 1).

Animal genetic selection is ideally no longer based on phenotypic measures but rather on estimated breeding values such as EPDs, which are estimated using best linear unbiased predictions in a multi-trait linear mixed model. Thus, the phenotypic (co)variance matrix is not needed for index construction [14,31]. Although the phenotypic correlations have no effect on the derivation of index weights (coefficients) they are required for the calculations describing the index [13]. As a second approach, where only EPDs are used, the only information needed in addition to the economic values to allow prediction of the breeding objective, is information on the genetic (co)variances among selection criteria in the index and on genetic (co)variances among the selection criteria and the breeding goals [14,32]. In that case, EPD was used instead of phenotypic measures in this index, and solving for the index coefficients is by an equation proposed by [14]: $b=G_{11}^{-1}G_{12}v$, where *b* is a vector of index weights for the EPD of the selection criteria in the index, *G*_*11*_ is a *n x n* genetic (co)variance matrix of the *n* selection criteria, *G*_*12*_ is a *n x m* genetic (co)variance matrix among the *n* selection criteria and the *m* breeding goals, and *v* is a vector of economic values, for which all values are the same (*v* = 1).

After estimating the weight indices, estimates of correlation among the indices were computed in order to analyze if the best animals selected with the multivariate approaches were the same as those identified based on the selection index proposed by [13] and [14]. Bayesian estimates of Spearman correlations between the indices were obtained using the package BayesianFirstAid [33].

## Results

The posterior marginal distributions of (co)variance component estimates were accurate, tending to normal distribution. The symmetrical distributions of measures of central tendency indicated accurate analysis [34,35]. In general, the samples obtained for the genetic correlations showed no wide dispersion (Tables 2–8), i.e. the oscillations remained stable, indicating that the burn-in period considered in the analysis was reliable and allowed convergence of the chain [36]. The estimates of genetic additive correlation among growth traits were all positive and high. Maternal genetic correlation estimates between the traits were all positive and ranging from 0.17 (BW-W210) to 0.96 (W120-W210).

**Table 2:** Marginal posterior distributions of the genetic direct, maternal, maternal permanent environmental, and residual variance components for growth and carcass traits in Polled Nellore cattle.

Trait	Mode	Mean	SD	Naive SE	Time series SE	CI 2.5%	CI 97.5%
	*Genetic additive direct*						
BW	3.724	3.784	0.243	0.00344	0.02181	3.34	4.29
W120	52.132	52.965	4.112	0.05816	0.50425	45.8	62.13
W210	140.072	137.789	7.809	0.11043	0.75908	123.5	153.4
W365	347.559	345.119	13.387	0.18932	0.89222	318.7	371.2
W450	369.652	366.457	15.922	0.22518	1.19783	336.3	398.6
LMA	12.167	12.192	1.101	0.01557	0.14785	10.12	14.36
BF	0.014	0.014	0.003	0.00004	0.00088	0.01	0.02
RF	0.081	0.078	0.009	0.00013	0.00142	0.06	0.1
	*Genetic additive maternal*						
BW	1.68	1.689	0.112	0.00158	0.01009	1.48	1.94
W120	22.681	23.726	2.108	0.0298	0.29554	19.26	27.88
W210	38.012	36.998	3.131	0.04427	0.41728	30.48	43.18
	Maternal permanent environmental						
BW	0.174	0.176	0.028	0.0004	0.005	0.12	0.24
W120	9.988	10.055	0.949	0.01342	0.10085	8.15	11.84
W210	14.82	14.876	1.646	0.02328	0.20409	11.87	18.2
	Residual						
BW	3.544	3.506	0.128	0.00181	0.01059	3.23	3.74
W120	128.601	127.533	2.544	0.03597	0.28408	122.2	132.1
W210	253.731	254.434	5.138	0.07266	0.42004	244.2	264.4
W365	280.249	281.285	9.036	0.12779	0.53815	263.6	299.2
W450	329.41	331.785	11.064	0.15647	0.8787	309.5	353.4
LMA	20.502	20.517	0.797	0.01127	0.09653	18.92	22.03
BF	0.134	0.134	0.003	0.00004	0.00061	0.13	0.14
RF	0.251	0.251	0.008	0.00011	0.00094	0.24	0.27

BW, birth weight; W120, weight at 120 days of age; W210, weight at 210 days of age; W365, weight at 365 days of age; W450, weight at 450 days of age; LMA: longissimus muscle area; BF: back-fat thickness; obtained between the 12th and 13th ribs; and RF: rump fat thickness; CI: Credibility Interval

**Table 3:** Marginal posterior distributions of the genetic covariances among growth and carcass traits in Polled Nellore cattle.

Trait		Mode	Mean	SD	Naive SE	Time series SE	CI 2.5%	CI 97.5%
		*Genetic additive direct*						
BW	W120	6.967	7.23	0.741	0.01049	0.07066	5.882	8.700
BW	W210	8.08	8.073	1.004	0.0142	0.08404	6.154	10.060
BW	W365	6.959	6.647	1.189	0.01681	0.07891	4.211	8.877
BW	W450	6.535	6.754	1.338	0.01892	0.09688	4.034	9.214
BW	LMA	0.479	0.559	0.368	0.00521	0.04081	0.018	1.244
BW	BF	0.017	0.009	0.02	0.00029	0.00335	0.003	0.045
BW	RF	-0.027	-0.027	0.04	0.00056	0.00559	-0.118	-0.005
W120	W210	78.151	80.786	5.333	0.07542	0.56556	71.220	92.371
W120	W365	107.247	107.801	6.188	0.08751	0.66018	96.350	120.400
W120	W450	107.465	109.464	6.672	0.09436	0.73264	96.690	123.303
W120	LMA	11.389	12.334	1.546	0.02186	0.19062	9.605	15.310
W120	BF	-0.009	-0.007	0.095	0.00135	0.02375	-0.150	0.000
W120	RF	-0.097	-0.099	0.163	0.0023	0.02535	-0.110	0.000
W210	W365	203.47	202.275	9.951	0.14073	0.84427	184.000	222.200
W210	W450	206.467	205.49	10.329	0.14607	0.8745	186.700	226.400
W210	LMA	24.227	24.18	2.225	0.03147	0.23896	19.750	28.440
W210	BF	0.134	0.103	0.143	0.00202	0.03323	0.016	0.323
W210	RF	-0.22	-0.26	0.257	0.00363	0.0395	-0.746	-0.028
W365	W450	354.369	350.976	13.681	0.19349	0.89219	324.400	378.800
W365	LMA	43.936	43.824	3.101	0.04385	0.29369	37.560	49.830
W365	BF	0.259	0.378	0.201	0.00284	0.0347	0.054	0.797
W365	RF	-0.565	-0.471	0.125	0.0046	0.04002	-0.910	-0.002
W450	LMA	43.709	43.602	3.211	0.04541	0.30801	37.310	49.600
W450	BF	0.521	0.52	0.19	0.00269	0.03377	0.192	0.923
W450	RF	-0.36	-0.324	0.344	0.00486	0.04371	-0.989	-0.039
LMA	BF	0.043	0.039	0.044	0.00062	0.00861	0.002	0.123
LMA	RF	-0.08	-0.089	0.077	0.00109	0.01143	-0.143	-0.001
BF	RF	0.016	0.018	0.004	0.00006	0.00078	0.010	0.026
		*Genetic additive maternal*						
BW	W120	1.418	1.615	0.393	0.00556	0.04942	0.837	2.46
BW	W210	1.385	1.267	0.45	0.00636	0.05562	0.389	2.204
W120	W210	28.543	28.452	2.454	0.03471	0.32642	23.19	33.09

BW, birth weight; W120, weight at 120 days of age; W210, weight at 210 days of age; W365, weight at 365 days of age; W450, weight at 450 days of age; LMA: longissimus muscle area; BF: back-fat thickness; obtained between the 12th and 13th ribs; and RF: rump fat thickness

**Table 4:** Marginal posterior distributions of the covariances among genetic additive direct and maternal effects among growth and carcass traits in Polled Nellore cattle.

Direct	Maternal	Mode	Mean	SD	Naive SE	Time series SE	CI 2.5%	CI 97.5%
BW	BW	-2.04	-2.034	0.144	0.00203	0.01252	-2.342	-1.776
BW	W120	-1.488	-1.274	0.519	0.00734	0.06464	-2.349	-0.186
BW	W210	-1.147	-1.15	0.598	0.00845	0.06946	-2.229	0
W120	BW	-2.095	-2.146	0.536	0.00758	0.06164	-3.221	-1.029
W120	W120	11.009	10.598	1.912	0.02703	0.26924	6.714	14.11
W120	W210	15.741	15.389	2.313	0.03271	0.31368	10.51	19.78
W210	BW	-1.453	-1.335	0.668	0.00945	0.05817	-2.671	-0.015
W210	W120	29.176	29.336	2.488	0.03519	0.26595	24.22	34.2
W210	W210	35.162	35.814	2.816	0.03982	0.26979	29.929	41.28
W365	BW	0.554	0.66	0.823	0.01164	0.0543	0.089	1.392
W365	W120	68.559	69.055	4.12	0.05826	0.39475	60.87	77.03
W365	W210	87.959	84.697	5.269	0.07451	0.53575	73.96	94.981
W450	BW	1.025	1.446	0.931	0.01317	0.06971	0.128	1.389
W450	W120	66.796	68.161	4.175	0.05905	0.39747	60.04	76.391
W450	W210	82.229	82.123	5.629	0.0796	0.61906	70.88	93.26
LMA	BW	-0.154	-0.073	0.27	0.00382	0.03671	-0.569	-0.052
LMA	W120	7.993	7.574	1.011	0.0143	0.13042	5.473	9.37
LMA	W210	9.401	9.696	1.278	0.01808	0.17997	7.128	12.12
BF	BW	0.007	0.006	0.013	0.00018	0.00205	0.002	0.031
BF	W120	0.089	0.06	0.057	0.00081	0.01041	0.004	0.178
BF	W210	0.064	0.078	0.079	0.00112	0.01605	0.007	0.23
RF	BW	0.015	0.009	0.029	0.00041	0.00432	0.004	0.075
RF	W120	-0.195	-0.144	0.107	0.00151	0.01588	-0.337	-0.062
RF	W210	-0.28	-0.199	0.127	0.00179	0.01783	-0.419	-0.017

BW, birth weight; W120, weight at 120 days of age; W210, weight at 210 days of age; W365, weight at 365 days of age; W450, weight at 450 days of age; LMA: longissimus muscle area; BF: back-fat thickness; obtained between the 12th and 13th ribs; and RF: rump fat thickness

**Table 5:** Marginal posterior distributions of the maternal permanent environmental and residual covariances among growth and carcass traits in Polled Nellore cattle.

Trait		Mode	Mean	SD	Naive SE	Time series SE	CI 2.5%	CI 97.5%
		*Maternal permanent environment*						
BW	W120	0.532	0.477	0.148	0.00209	0.02729	0.164	0.764
BW	W210	0.457	0.382	0.198	0.00281	0.0377	0.019	0.802
W120	W210	11.51	11.827	1.208	0.01709	0.13657	9.466	14.21
		*Residual*						
BW	W120	4.56	4.706	0.413	0.00584	0.03483	3.911	5.486
BW	W210	6.009	5.998	0.578	0.00817	0.04495	4.855	7.117
BW	W365	6.725	6.799	0.685	0.00969	0.03848	5.478	8.204
BW	W450	7.728	7.542	0.783	0.01107	0.04514	6.07	9.122
BW	LMA	0.408	0.538	0.217	0.00307	0.01837	0.119	0.968
BW	BF	0.024	0.021	0.014	0.0002	0.00174	0.007	0.049
BW	RF	0.033	0.029	0.025	0.00035	0.00249	0.02	0.078
W120	W210	148.59	148.919	3.383	0.04785	0.3184	141.9	155.1
W120	W365	134.7	133.447	3.942	0.05574	0.30359	125.8	141.203
W120	W450	127.885	128.829	4.395	0.06215	0.33936	120.1	137.4
W120	LMA	17.055	16.889	1.164	0.01646	0.11777	14.58	19.1
W120	BF	0.276	0.311	0.08	0.00113	0.01187	0.151	0.462
W120	RF	0.193	0.164	0.123	0.00174	0.01388	0.089	0.398
W210	W365	213.159	213.769	6.355	0.08987	0.50626	201.198	226.2
W210	W450	202.326	200.672	6.777	0.09584	0.52954	187.198	213.9
W210	LMA	24.553	24.753	1.732	0.0245	0.15033	21.33	28.091
W210	BF	0.582	0.55	0.122	0.00172	0.0182	0.298	0.779
W210	RF	0.378	0.402	0.196	0.00277	0.02177	0.013	0.773
W365	W450	255.158	257.001	9.283	0.13128	0.5337	238.7	275.103
W365	LMA	28.808	29.775	2.286	0.03233	0.20738	25.35	34.34
W365	BF	0.744	0.723	0.162	0.00229	0.02277	0.399	1.02
W365	RF	0.997	1.02	0.245	0.00347	0.0222	0.525	1.498
W450	LMA	40.961	40.098	2.417	0.03417	0.21669	35.5	44.87
W450	BF	0.914	0.896	0.163	0.00231	0.01701	0.576	1.209
W450	RF	1.496	1.525	0.264	0.00373	0.02609	0.997	2.04
LMA	BF	0.273	0.282	0.035	0.0005	0.00559	0.215	0.358
LMA	RF	0.439	0.435	0.058	0.00082	0.00735	0.327	0.555
BF	RF	0.058	0.058	0.003	0.00005	0.00056	0.051	0.064

BW, birth weight; W120, weight at 120 days of age; W210, weight at 210 days of age; W365, weight at 365 days of age; W450, weight at 450 days of age; LMA: longissimus muscle area; BF: back-fat thickness; obtained between the 12th and 13th ribs; and RF: rump fat thickness

**Table 6:** Marginal posterior distributions of the genetic correlation among the growth and carcass traits in Polled Nellore cattle.

Trait		Mode	Mean	SD	Naive SE	Time series SE	CI 2.5%	CI 97.5%
		*Genetic additive direct*						
BW	W120	0.506	0.511	0.041	0.00058	0.00479	0.427	0.587
BW	W210	0.342	0.354	0.04	0.00056	0.00371	0.274	0.428
BW	W365	0.187	0.184	0.032	0.00046	0.00222	0.117	0.244
BW	W450	0.193	0.181	0.035	0.0005	0.00265	0.109	0.243
BW	LMA	0.077	0.083	0.054	0.00077	0.00622	0.003	0.184
BW	BF	0.083	0.042	0.092	0.0013	0.01586	0.001	0.208
BW	RF	-0.028	-0.049	0.074	0.00105	0.01061	-0.216	-0.001
W120	W210	0.953	0.946	0.011	0.00015	0.00274	0.924	0.964
W120	W365	0.781	0.798	0.026	0.00037	0.00404	0.751	0.848
W120	W450	0.799	0.786	0.031	0.00044	0.00537	0.729	0.844
W120	LMA	0.473	0.487	0.057	0.0008	0.00896	0.384	0.595
W120	BF	-0.011	0.043	0.115	0.00163	0.02954	-0.17	-0.001
W120	RF	-0.048	-0.049	0.08	0.00114	0.01279	-0.198	-0.001
W210	W365	0.927	0.928	0.012	0.00018	0.00242	0.899	0.949
W210	W450	0.925	0.915	0.019	0.00027	0.00468	0.872	0.945
W210	LMA	0.585	0.591	0.043	0.0006	0.00577	0.505	0.669
W210	BF	0.095	0.08	0.109	0.00154	0.02674	0.001	0.329
W210	RF	-0.061	-0.079	0.078	0.00111	0.01222	-0.125	-0.003
W365	W450	0.988	0.987	0.003	0.00004	0.00078	0.98	0.991
W365	LMA	0.679	0.676	0.029	0.00041	0.00323	0.616	0.733
W365	BF	0.117	0.176	0.095	0.00134	0.01789	0.024	0.378
W365	RF	-0.094	-0.091	0.064	0.0009	0.00802	-0.214	-0.001
W450	LMA	0.652	0.653	0.029	0.0004	0.00287	0.595	0.709
W450	BF	0.235	0.235	0.086	0.00122	0.01549	0.085	0.413
W450	RF	-0.058	-0.061	0.065	0.00092	0.0084	-0.187	-0.001
LMA	BF	0.086	0.095	0.11	0.00156	0.02314	0.002	0.309
LMA	RF	-0.092	-0.092	0.08	0.00114	0.01243	-0.261	-0.001
BF	RF	0.523	0.547	0.074	0.00104	0.01528	0.378	0.683
		*Genetic additive maternal*						
BW	W120	0.25	0.254	0.055	0.00078	0.00737	0.136	0.362
BW	W210	0.171	0.16	0.054	0.00077	0.00663	0.05	0.263
W120	W210	0.966	0.96	0.012	0.00016	0.00327	0.939	0.979

BW, birth weight; W120, weight at 120 days of age; W210, weight at 210 days of age; W365, weight at 365 days of age; W450, weight at 450 days of age; LMA: longissimus muscle area; BF: back-fat thickness; obtained between the 12th and 13th ribs; and RF: rump fat thickness

**Table 7:** Marginal posterior distributions of the correlation between genetic additive direct and maternal correlation among the growth and carcass traits in Polled Nellore cattle.

Direct	Maternal	Mode	Mean	SD	Naive SE	Time series SE	CI 2.5%	CI 97.5%
BW	BW	-0.807	-0.804	0.017	0.00024	0.00151	-0.835	-0.769
BW	W120	-0.149	-0.134	0.052	0.00073	0.00634	-0.234	-0.022
BW	W210	-0.095	-0.096	0.049	0.00069	0.00566	-0.184	-0.002
W120	BW	-0.228	-0.227	0.054	0.00076	0.00648	-0.326	-0.110
W120	W120	0.267	0.302	0.066	0.00093	0.01105	0.179	0.425
W120	W210	0.323	0.350	0.059	0.00083	0.00911	0.231	0.463
W210	BW	-0.099	-0.087	0.043	0.0006	0.00368	-0.169	-0.001
W210	W120	0.527	0.515	0.049	0.00069	0.00744	0.414	0.600
W210	W210	0.495	0.503	0.044	0.00062	0.00594	0.407	0.589
W365	BW	0.024	0.027	0.034	0.00048	0.00222	0.004	0.098
W365	W120	0.777	0.764	0.026	0.00036	0.00347	0.709	0.811
W365	W210	0.753	0.750	0.029	0.0004	0.00431	0.694	0.807
W450	BW	0.044	0.058	0.037	0.00052	0.00277	0.001	0.135
W450	W120	0.726	0.732	0.029	0.00042	0.00408	0.674	0.785
W450	W210	0.709	0.706	0.033	0.00047	0.00516	0.637	0.771
LMA	BW	0.004	0.016	0.060	0.00085	0.00816	0.001	0.114
LMA	W120	0.472	0.446	0.054	0.00076	0.00787	0.337	0.545
LMA	W210	0.431	0.457	0.053	0.00075	0.00796	0.347	0.554
BF	BW	0.052	0.038	0.085	0.0012	0.01385	0.001	0.216
BF	W120	0.157	0.159	0.099	0.0014	0.0185	0.071	0.296
BF	W210	0.204	0.198	0.110	0.00156	0.02311	0.110	0.297
RF	BW	0.051	0.024	0.081	0.00114	0.01203	0.001	0.106
RF	W120	-0.098	-0.106	0.079	0.00112	0.01213	-0.250	-0.005
RF	W210	-0.158	-0.118	0.076	0.00108	0.01119	-0.254	-0.003

BW, birth weight; W120, weight at 120 days of age; W210, weight at 210 days of age; W365, weight at 365 days of age; W450, weight at 450 days of age; LMA: longissimus muscle area; BF: back-fat thickness; obtained between the 12th and 13th ribs; and RF: rump fat thickness

**Table 8:** Marginal posterior distributions of the genetic additive direct and maternal heritability, and proportion of phenotypic variance due to maternal permanent environmental effects for growth and carcass traits in Polled Nellore cattle.

Trait	Mode	Mean	SD	Naive SE	Time series SE	CI 2.5%	CI 97.5%
	*Direct heritability*						
BW	0.527	0.531	0.030	0.00042	0.00270	0.48	0.59
W120	0.231	0.235	0.017	0.00024	0.00220	0.20	0.27
W210	0.289	0.287	0.015	0.00021	0.00158	0.26	0.32
W365	0.552	0.551	0.017	0.00024	0.00111	0.52	0.58
W450	0.528	0.525	0.018	0.00026	0.00136	0.49	0.56
LMA	0.370	0.372	0.029	0.00041	0.00388	0.32	0.43
BF	0.073	0.093	0.020	0.00028	0.00580	0.06	0.14
RF	0.227	0.237	0.027	0.00038	0.00427	0.19	0.29
	*Maternal heritability*						
BW	0.238	0.237	0.014	0.00020	0.00130	0.21	0.27
W120	0.106	0.105	0.009	0.00013	0.00140	0.09	0.12
W210	0.080	0.077	0.006	0.00009	0.00088	0.06	0.09
	Maternal permanent environmental proportion (c²)						
BW	0.022	0.025	0.004	0.00006	0.00072	0.02	0.03
W120	0.044	0.045	0.004	0.00006	0.00046	0.04	0.05
W210	0.031	0.031	0.003	0.00005	0.00043	0.02	0.04

BW, birth weight; W120, weight at 120 days of age; W210, weight at 210 days of age; W365, weight at 365 days of age; W450, weight at 450 days of age; LMA: longissimus muscle area; BF: back-fat thickness; obtained between the 12th and 13th ribs; and RF: rump fat thickness

The correlations between weights at birth and the other weight categories were the only genetic correlations that presented negative values regarding the maternal effects, which means that selection of the best cows cannot result in selection of the best progenies (Table 3). Genetic correlation among birth weights and the weights at 120, 210, 365 and 450 days of age were all positive (Table 4).

Additive direct heritabilities for weights at birth, 120, 210, 365 and 450 days of age, longissimus muscle area, back-fat thickness, and rump fat thickness were 0.53±0.030, 0.24±0.017, 0.29±0.015, 0.55±0.017, 0.53±0.018, 0.37±0.029, 0.09±0.020 and 0.24±0.027, respectively. The estimates of maternal heritabilities were 0.24±0.014, 0.11±0.009 and 0.08±0.006, respectively for weights at birth, 120, 210 days of age (Table 8). Estimates were moderate and remained fairly similar within pre-weaning traits and within post-weaning traits. The estimates of maternal permanent environmental effects (c^2^) for weights at birth, 120 and 210 days of age were 0.025±0.004, 0.045±0.004 and 0.031±0.003, respectively, which means that repeatability of the dam in this herd did not affect the expression of these traits, or on the other hand, the dams were constantly changing.

**Fig 1:**
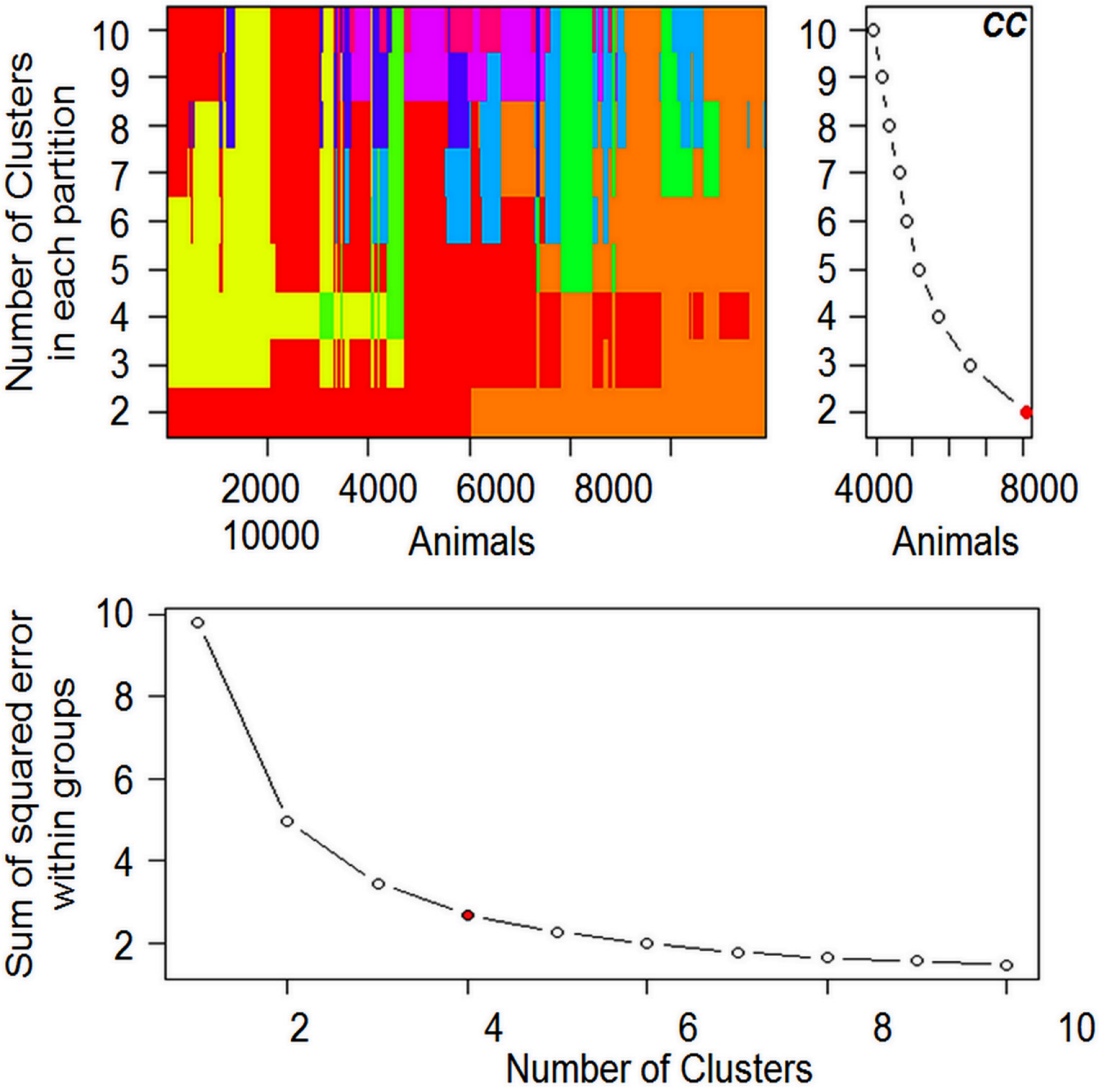
Plots of different approaches (visual k-mean partition comparison, Calinsky criterion (*CC*), and sum of squared error within groups) used in order to detect the optimal number of clusters. Using the k-mean comparison, the number of colors is in accordance with the number of clusters in each partition.

Before performing cluster analysis, it was necessary to diagnose how many clusters suited the data. Thus, visual examination were used to determine the best k value: the first consisted of using the Calinsky criterion (*CC*) technique; and the second approach was the sum of squared errors (SSE) within groups (Fig 1). In accordance with the k-mean partition comparison and the *CC*, the best k was 2, and for the SSE within groups the best k was 4; therefore we used the mean of both approaches (k = 3).

After performing cluster analysis using the k-mean method and k = 3, groups 1, 2 and 3 were composed of 2970, 5886 and 2997 animals. Significant differences were observed between the three groups (p < 0.0001), as pointed out in Table 8. It was possible to sort the animals that had similar EPDs into each cluster. Kruskal-Wallis rank sum test, followed by Dunn’s test and p-value adjusted by FDR analysis revealed differences among these groups for weights and carcass traits. Thus, using this approach it is possible to choose the best animals for selection, or conversely choose inferior animals to cull. Cluster analyses allow one to perform more accurate culling of the worst animals, meaning those that do not contribute positively in the herd, as well as helping to select the best animals.

**Fig 2:**
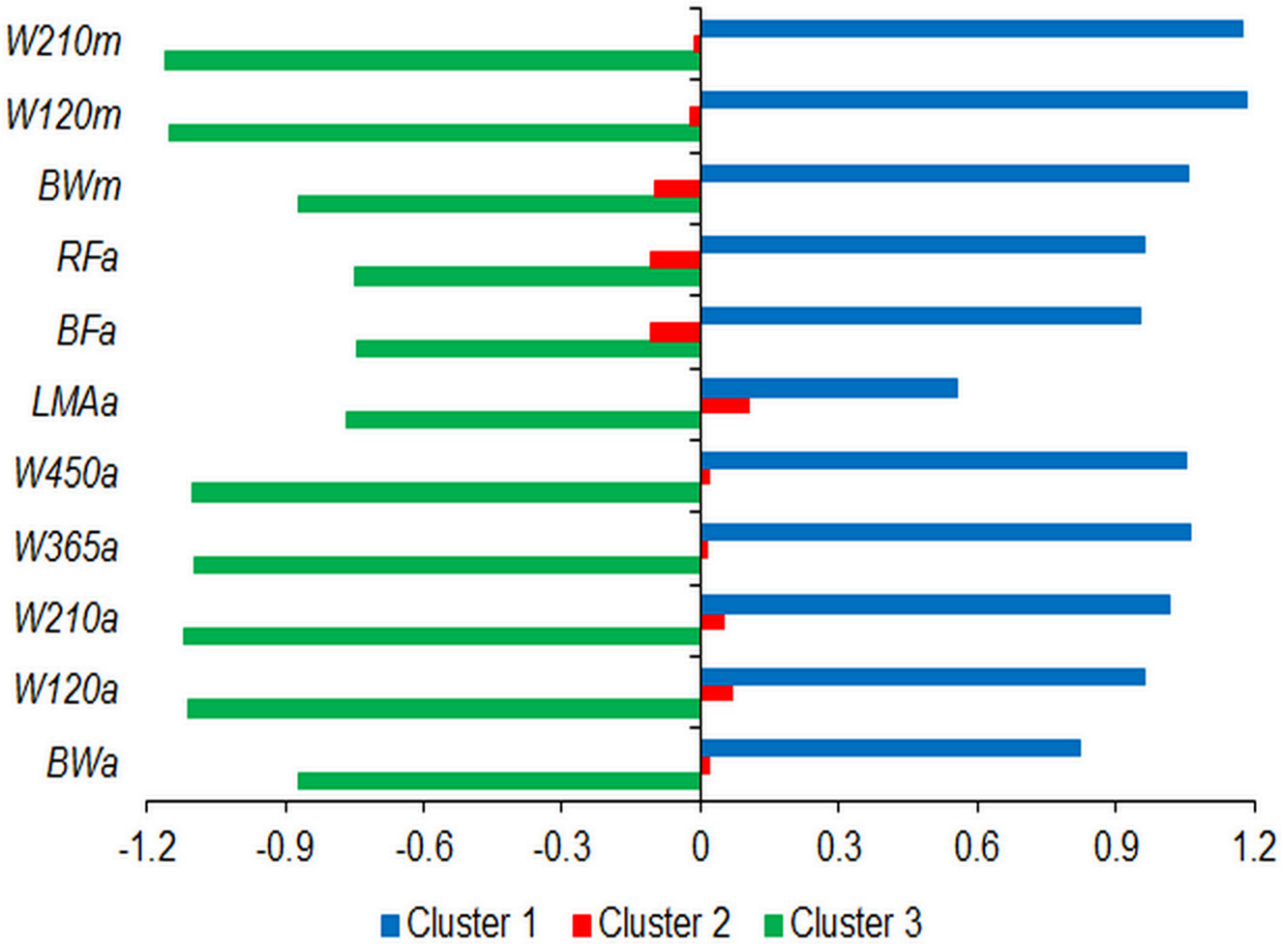
Standardized average of direct (*a*) and maternal (*m*) expected progeny difference for growth and carcass traits in each cluster. BW, birth weight; W120, weight at 120 days of age; W210, weight at 210 days of age; W365, weight at 365 days of age; W450, weight at 450 days of age; LMA: longissimus muscle area; BF: back-fat thickness; obtained between the 12th and 13th ribs; RF: rump fat thickness.

The K-mean method was efficient in grouping animals based on similar EPDs, and its standardized averages are shown in Fig 2. The animals grouped in cluster 1 had the highest EPDs for all traits, cluster 2 presented average animals, and cluster 3 comprised the animals with the lowest EPDs. There were significant difference between these groups for all traits (Table 9). Thus, this approach allowed a simulation in order to choose the superior animals for selection and the inferior animals for culling.

**Table 9:** Kruskal-Wallis rank sum test and Dunn’s test for multiple pairwise comparisons of expected progeny differences for weights and carcass traits among the clusters.

Trait	DF	*χ*^2^	p-value
**Direct effect**			
*Birth Weight*	2	4400.0173	***
*Weight at 120 days*	2	7034.7213	***
*Weight at 210 days*	2	7443.3853	***
*Weight at 365 days*	2	7678.1952	***
*Weight at 450 days*	2	7711.2914	***
*Longissimus muscle area*	2	2645.4559	***
*Back-fat thickness*	2	4703.2899	***
*Rump fat thickness*	2	4736.5479	***
**Maternal effect**			
*Birth Weight*	2	5807.0458	***
*Weight at 120 days*	2	8748.0507	***
*Weight at 210 days*	2	8841.5603	***

DF: degrees of freedom; *χ*^2^: Kruskal-Wallis chi-squared

*** (p < 0.0001)

The linear discriminant coefficients LD1 and PC1 were established as the two equations that allowed for the best discrimination (Table 10). The proportion of trace indicates the amount of data for which each equation can successfully account, regarding standardized EPDs. The first linear discriminant function (LD1) was used because its proportion of trace was close to 1 (0.9837). The accuracy of the selection index was 0.969. The index weights, as proposed by [12] and [14], have only values for measurable traits, in other words, only for phenotypes. Estimates of correlations between pSI-LD1 (0.66±0.061), pSI-PC1 (0.66±0.061), pSI-gSI (0.64±0.066), LD1-PC1 (0.99±0.0001), LD1-gSI (0.96±0.0094) and PC1-gSI (0.96±0.0085) were positive, and their values have magnitudes from moderate to high (Table 11).

**Table 10:** Weights of indices based on selection index and the multivariate approach.

Index	Direct Effect								Maternal Effect		
	BW	W120	W210	W365	W450	LMA	BF	RF	BW	W120	W210
gSI	0.802	1.110	1.181	0.418	0.576	0.632	1.505	-0.748			
pSI	0.794	0.067	-0.080	0.090	-0.029	0.065	2.165	1.287			
LD1	0.410	0.424	0.434	0.387	0.390	0.265	0.131	0.145	0.076	0.142	0.164
PC1	-1.241	-2.818	-3.359	4.473	3.241	1.645	0.179	0.044	0.926	2.265	1.789

pSI: Selection Index proposed by Hazel (1943); gSI: Selection Index proposed by Schneeberger et al. (1992); LD1: first linear discriminant function; PC1: first principal component; BW, birth weight; W120, weight at 120 days of age; W210, weight at 210 days of age; W365, weight at 365 days of age; W450, weight at 450 days of age; LMA: longissimus muscle area; BF: back-fat thickness; obtained between the 12th and 13th ribs; RF: rump fat thickness

**Table 11:** Mean, standard deviation (SD), naive standard error (SE) of the mean and time-series standard error (SE) of Spearman correlation among the predicted values from selection index and multivariate approaches.

	Mean	SD	Naive SE	Time-series SE
pSI x LD1	0.66	0.0612	0.0004999	0.0009603
pSI x PC1	0.66	0.0610	0.0004983	0.0009955
pSI x gSI	0.63	0.0658	0.0005377	0.0010620
LD1_PC1	0.99	0.0001	0.0000009	0.0000107
LD1 x gSI	0.96	0.0093	0.0000764	0.0002856
PC1 x gSI	0.96	0.0085	0.0000693	0.0002904

pSI: Selection Index proposed by Hazel (1943); gSI: Selection Index proposed by Schneeberger et al. (1992); LD1: first linear discriminant function; PC1: first principal component

**Fig 3:**
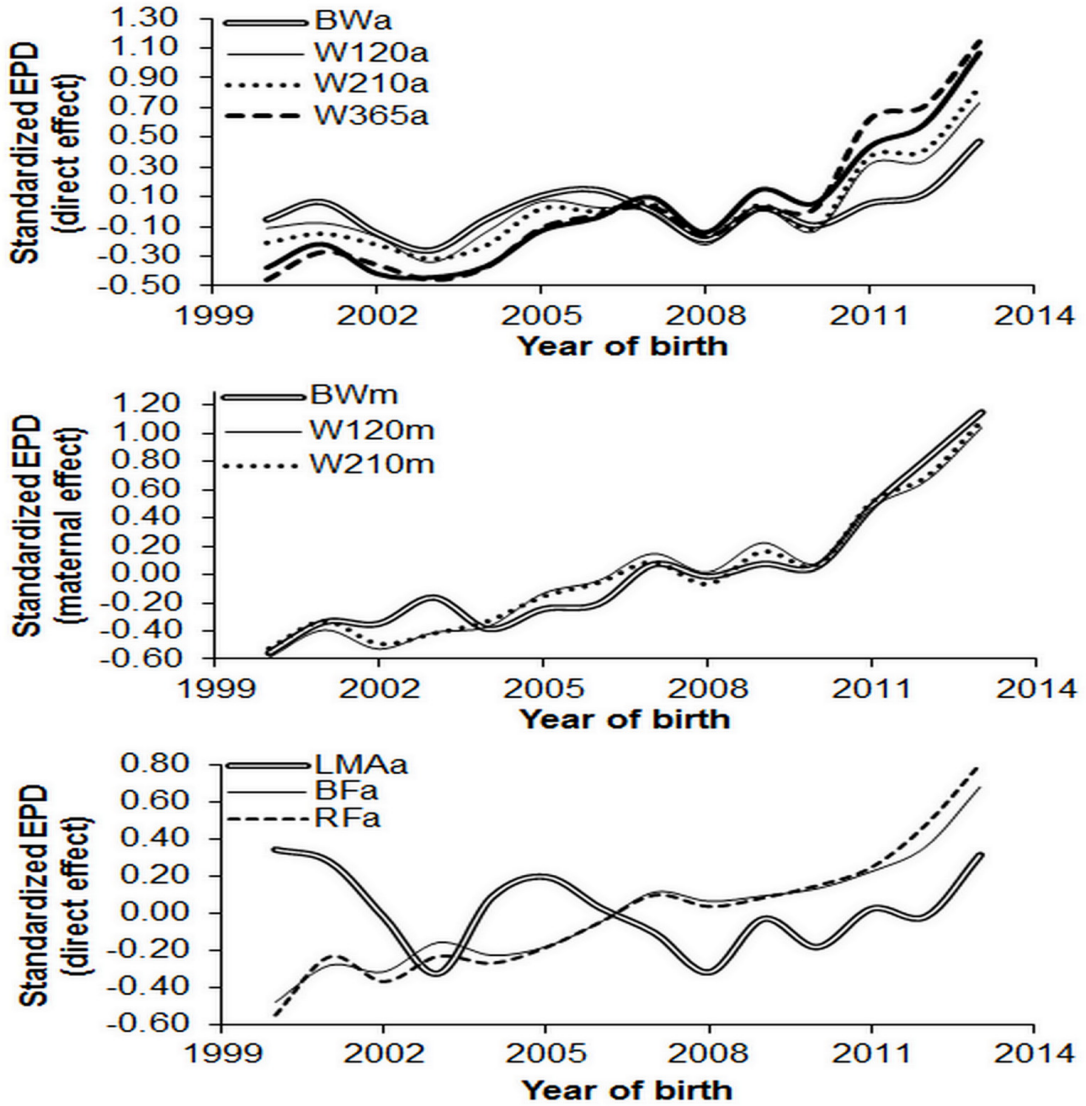
Standardized genetic trends for weights (BW, W120, W210, W365 and W450) and carcass (LMA, BF and RF) traits due to direct and maternal effects. BW, birth weight; W120, weight at 120 days of age; W210, weight at 210 days of age; W365, weight at 365 days of age; W450, weight at 450 days of age; LMA: longissimus muscle area; BF: back-fat thickness; obtained between the 12th and 13th ribs; RF: rump fat thickness.

**Fig 4:**
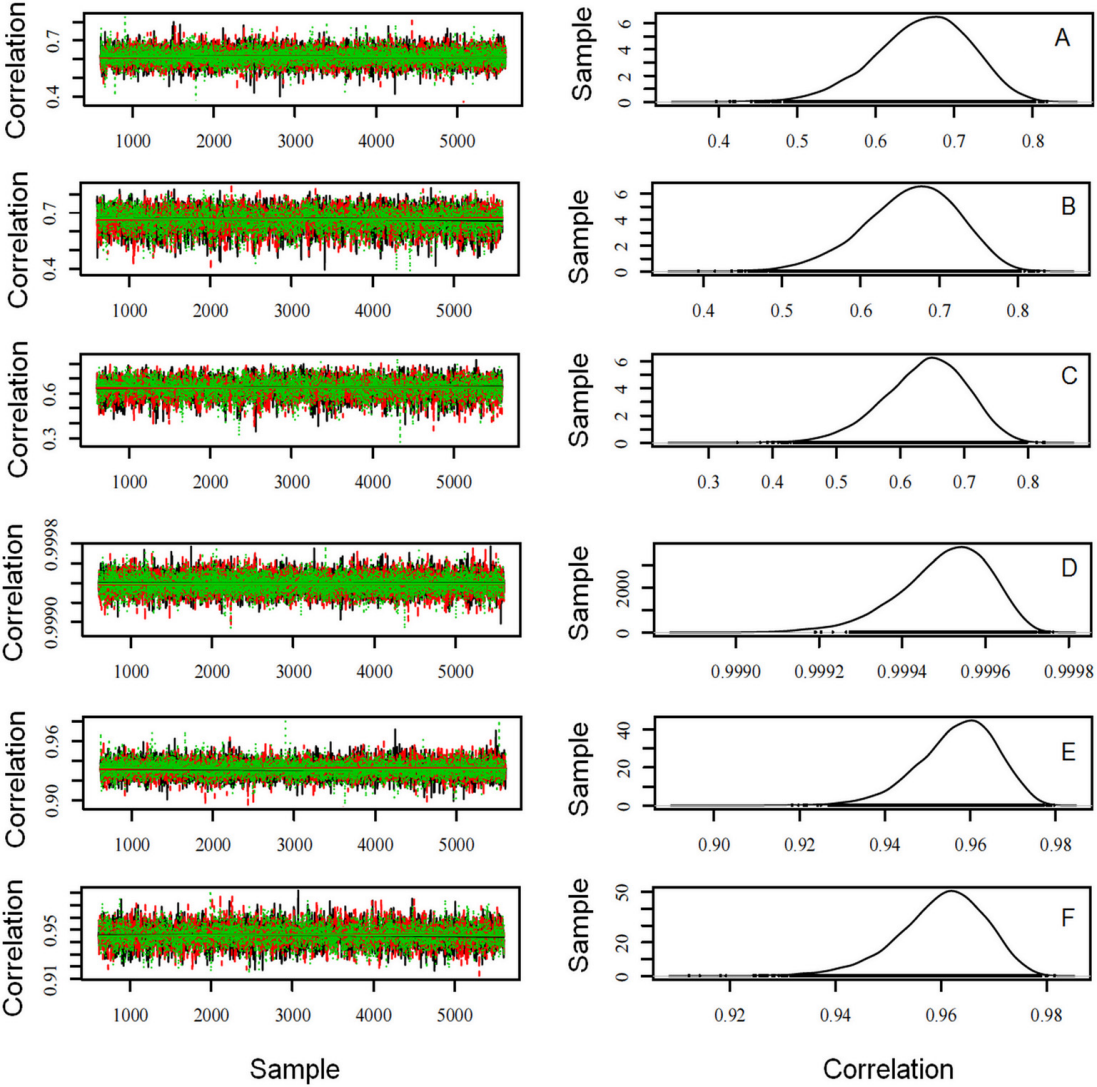
Estimates of correlation between indices pSI-LD1 (A), pSI-PC1 (B), pSI-gSI (C), LD1-PC1 (D), LD1-gSI (E) and PC1-gSI (F). pSI: Selection Index proposed by [13]; gSI: Selection Index proposed by [14]; LD1: first linear discriminant function; and PC1: first principal component.

Favorable genetic trends were obtained for the direct and maternal standardized EPD for birth weight, weight at 120, 210, 365 and 450 days of age, back-fat thickness, and rump fat thickness; except for longissimus muscle area which showed sigmoidal trend (Fig 3). Trace and density plots of marginal posterior Spearman correlations (Fig 4) between the selection indexes proposed by [13] and [14], first linear discriminant function, and the first principal component were accurate, tending to normal distribution, and showing symmetrical distributions of measures of central tendency, which indicate high accuracy in these analyses [34,37].

## Discussion

Moderate to high estimates of direct heritabilities for growth and carcass traits suggest a gap for genetic improvement of the traits using mass selection [10, 38–42]. Overall, the estimate of maternal genetic effects was moderate and affirmed the importance of maternal genetics in the expression of the phenotype of Nellore cattle regarding growth traits. Estimates of maternal permanent environmental effects (c^2^) were quite low, but enough to influence the expression of an individual’s real phenotype due to masking effects of pre-weaning care. This indicates a higher effect of the maternal permanent environmental effect on weaning weight and a declining trend in the subsequent weight categories.

The genetic correlations between direct additive genetic and maternal additive genetic effects were negative only between correlations regarding birth weight. Therefore, selection for direct additive genetic effects would not improve maternal ability, making it difficult to conduct joint selection for these traits. Different reasons for these negative estimates have been proposed. As reported by [43], the number of offspring per dam may have increased the dependence between maternal parameters, although other factors such as the number of dams with records also interfere in these estimates. This occurs due to failure to include some important fixed effects in the model, and the inclusion of sire x year interaction in the model could also lead to a reduction in the negative correlation estimates between the animal effects [11]. The negative correlations may also indicate antagonism between the effects of genes related to growth and maternal ability and are often considered to be a statistical issue rather than a biological matter in animal breeding [44, 45]. Antagonism between the effects of an individual’s genes for growth and those of its dam for a maternal contribution may also be due to natural selection for an intermediate optimum [46]. On the other hand, estimates of genetic correlation between direct and maternal effects for pre and post weaning weights (W120, W210, W365 and W450) were all positive, which means that the selection for direct genetic effects can improve maternal ability, enabling joint selection for growth traits.

Estimates of direct heritabilities were moderate to high for all traits, suggesting that genetic improvement can be obtained through the selection for these traits. Estimates of direct heritabilities for post weaning live weights previously reported by [41] were similar to the estimates obtained in the present study. The estimates of genetic correlations between genetic direct additive and maternal additive effects were moderate and were negative only between BW and the other weights, which reflected an antagonistic relationship. This antagonistic effect is hard to explain and probably occurred for the reasons cited above.

The estimates of genetic correlations among all traits were positive and ranged from moderate to high, indicating strong genetic associations among all traits, in accordance with results reported by [4,40,41] regarding Nellore cattle. Genetic correlations between weaning weight and post weaning weights were also moderate to high and positive, suggesting that many of the genetic factors that influence body weight at different ages were the same and that selection of animals at weaning age is associated with the weight at 15 months. Likewise, high genetic correlations of W120 with other traits mean that animals presenting greater EPDs for W210 will probably present greater genetic merit for W365 and W450 also. Therefore, reduction of the age of selection from 450 days to 365 days or even at weaning (W210) may be possible for Nellore cattle.

High genetic variability observed in the herd, indicated by high estimates of the heritability coefficients, reveal that it is possible to make improvements over generations through a selection schedule [2,47,48] aiming at genetic progress of traits. Genetic trends (Fig 3) were positive and increasing over the years, which may have contributed to the correct classification and grouping of similar animals within each cluster.

Animals in cluster 1 presented the highest values when the selection indices were used, which points to similar results regardless of the approach adopted to select superior animals. This statement is supported by positive and high correlation estimates between the first linear discriminant (LD1) function and the other selection indices [13]. Therefore, in the absence of economic weights, the multivariate approach can be a reliable tool for the purpose of selecting the best animals. Multivariate analysis allowed forecasting information based on the relationships among breeding values and accounting for maternal ability and the direct genetic potential of the animals. Likewise, the multivariate index enabled data summarization after genetic evaluation, fine discrimination and rapid selection of animals.

The results of this study contribute to achieving the goals of simplifying the process of cattle breeding, lowering costs and saving time to subsidize on-farm decision-making regarding the selection of sires and dams. The generation of progeny with superior genetic merit for traits of economic importance is crucial to increase profitability in beef cattle production. Currently, economic returns are obtained mainly by selling heavier animals for slaughtering, with a growing emphasis on carcass quality. This logic seems to perfectly match the increased genetic trends found in this study.

The selection index approach is limited due to working only with phenotypes (selection criteria), requiring farm-specific economic and productive data and needing a separate index for every animal. These costly and time-consuming limitations are overcome by using the multivariate technique presented and tested in this study. Nevertheless, despite some limitations, the economic selection approach to weights and carcass traits is no doubt the most robust method for the selection of cattle.

## Conclusion

Genetic correlations were substantial across in traits of economic importance evaluated in this study. High to moderate estimates of heritabilities and genetic correlations indicate that a correlated response approach appears to be suitable for practical decision-making in beef cattle systems. The results obtained from the multivariate approach were consistent with results obtained using selection indices, which supports the use of multivariate analysis as a potential tool for selection in cattle breeding. In addition, longissimus muscle area and subcutaneous fat thickness measured by real-time ultrasound should be used as selection criteria, allowing the estimation of breeding values before the first mating season in order to accelerate the response to individual selection.
